# Thermo-Mechanical Behavior of Poly(ether ether ketone): Experiments and Modeling

**DOI:** 10.3390/polym13111779

**Published:** 2021-05-28

**Authors:** A. D. Drozdov, J. deClaville Christiansen

**Affiliations:** Department of Materials and Production, Aalborg University, Fibigerstraede 16, 9220 Aalborg, Denmark; jc@mp.aau.dk

**Keywords:** poly(ether ether ketone), thermo–mechanical response, constitutive modeling

## Abstract

Observations are reported on poly(ether ether ketone) (PEEK) in uniaxial tensile tests, relaxation tests and creep tests with various stresses in a wide interval of temperatures ranging from room temperature to 180 °C. Constitutive equations are developed for the thermo–mechanical behavior of PEEK under uniaxial deformation. Adjustable parameters in the governing equations are found by matching the experimental data. Good agreement is demonstrated between the observations and results of numerical simulation. It is shown that the activation energies for the elastoplastic, viscoelastic and viscoelastoplastic responses adopt similar values at temperatures above the glass transition point.

## 1. Introduction

Poly(ether ether ketone) (PEEK) is a semicrystalline thermoplastic homopolymer with a linear molecular structure and relatively stiff backbone chains. This polymer belongs to the family of poly(ether ketones) whose ethers functional groups are linked together through aromatic groups. PEEK is a high performance polymer that displays a unique combination of toughness, stiffness, strong abrasion resistance and tribological performance, low moisture absorption, thermo-oxidative stability, chemical and solvent resistance, biocompatibility, flame retardancy, and retention of physical properties at elevated (up to 200 °C) temperatures [1]. Due to the excellent balance of mechanical and physical properties, this polymer and composites with PEEK matrices are widely used in aerospace [2,3] and automobile industries [4], energy technologies [5,6] and biomedicine [7,8].

Due to the importance of mechanical properties for application of poly(ether ether ketone) as a load-bearing material, a number of studies have dealt with the experimental investigation of the thermo–mechanical response of PEEK. Results of DMA analysis in the temperature-sweep mode are presented in [9,10,11,12,13,14]. Observations in tests with various strain rates are reported in [10,15,16,17,18,19] for tensile deformation, in [10,20,21,22] for compressive deformation, and in [23,24] for biaxial loading. Experimental data in cyclic and fatigue tests are presented in [10,14,25,26,27,28,29]. Observations in the Izod impact tests are given in [13,14,30], whereas those in the Taylor impact tests are provided in [31,32,33]. Observations in nanoindentation tests are discussed in [34,35]. Experimental data in relaxation tests are reported in [11,36,37], and those in creep tests are presented in [11,38,39,40].

As PEEK is a semicrystalline polymer, its time- and rate-dependent behavior can be described by conventional models in viscoelasticity and viscoplasticity of semicrystalline polymers, see [41,42,43], to mention a few. Constitutive equations accounting for the peculiarities in the thermo–mechanical response of PEEK induced by stiffness of its backbone chains were developed in [18,19,22,24,29,44,45,46].

Previous studies on the thermo–mechanical behavior of PEEK focused on its viscoelastic and viscoplastic responses below the glass transition temperature $T_{c}\approx 150$ °C [24,29,44,45,46]. Above this temperature, only observations in tensile tests with constant strain rates were reported and analyzed in [18,19,22]. The mechanical behavior of PEEK and its micro- and nanocomposites at temperatures exceeding $T_{c}$ have recently attracted substantial attention due to applications of sulfonated PEEK-based polymers as membrane materials in polymer electrolyte membrane fuel cells and direct methanol fuel cells with the interval of working temperatures up to 180 °C [47,48,49]. The aim of this study is to perform a thorough investigation of the mechanical behavior of PEEK both below and above its glass transition temperature. In particular, we concentrate on the activation energies for the viscoelastic and viscoplastic processes in the sub-$T_{g}$ and post-$T_{g}$ intervals. These characteristics allow correlations to be established between mobility of chains and the structure of polymer networks, on the one hand, and physical properties of PEEK membranes in high-temperature electrochemical cells (ionic conductivity, methanol permeability, dielectric permittivity, thermal stability, chemical resistance), on the other [50].

The objective of this paper is three-fold: (i) to analyze experimentally the thermo-mechanical response of PEEK in uniaxial tensile tests with a constant strain rate, relaxation tests with a constant strain, and creep tests with various stresses in a wide interval of temperatures from room temperature up to 180 °C, (ii) to develop constitutive equations for the thermo–elastoplastic, thermo–viscoelastic and thermo–viscoelastoplastic responses of PEEK and to find material constants in these relations by matching the observations, and (iii) to compare activation energies for the elastoplastic behavior (tensile tests with small strains), viscoelastic response (short-term relaxation tests and creep tests in the linear regime of deformation) and viscoelastoplastic behavior (creep tests with relatively large stresses above the glass transition temperature).

Unlike previous studies on modeling the time- and rate-dependent behavior of PEEK subjected to arbitrary 3D deformations with finite strains [19,24,29,44,46], we confine ourselves to the analysis of its thermo-mechanical response under uniaxial deformation with small strains. This allows the number of adjustable coefficients in the constitutive equations to be reduced noticeably (compared with conventional models). As a result, the effect of temperature on the material parameters can be determined with high accuracy. The latter is of primary importance for (i) the design of PEEK implants produced by additive manufacturing, 3D printing and fused deposition modeling (FDM) technology, and (ii) prediction of their microstructure, tribological performance and mechanical properties [51,52,53].

## 2. Materials and Methods

Poly(ether ether ketone) KETRON 1000 PEEK FKM NATUR (density 1.31 g/cm${}^{3}$, tensile modulus 4.34 GPa, ultimate tensile strength 110 MPa) was supplied as extruded sheets by Vink Plast ApS (Denmark). Dumbbell specimens for tensile tests (ASTM standard D638) with length in the active zone 50 mm, width 5.1 mm, and thickness 4.5 mm were machined from the sheet. To exclude the effect of stresses developed under preparation, tests were conducted a week after machining samples.

**Figure 1:**
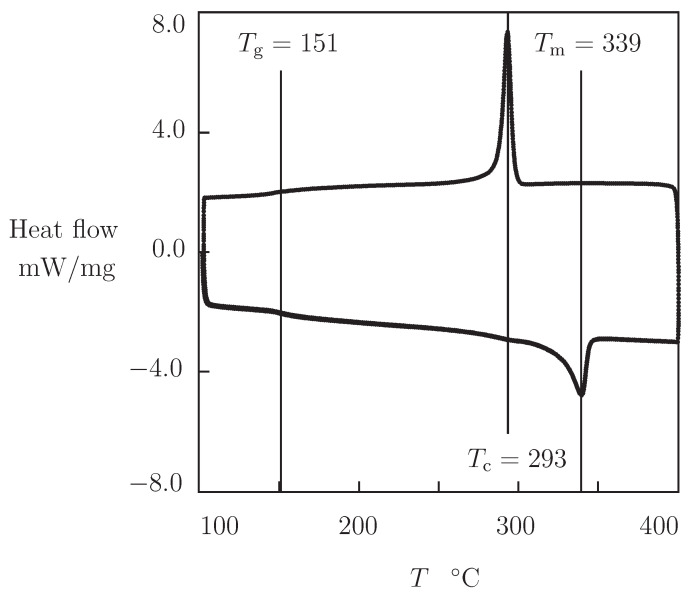
DSC thermogram of PEEK. Solid line: experimental data in DSC test with the heating and cooling rates of 20 K/min.

Differential scanning calorimetry (DSC) measurements were carried out by means of STA 449/Netzsch apparatus at the heating and cooling rate of 20 K/min. Specimens with mass of about 10 mg were tested in alumina pans covered by lid under argon atmosphere. The experimental program involves: heating from the initial temperature $T_{i}=104$ up to the final temperature $T_{f}=400$ °C, followed by cooling to the initial temperature $T_{i}$, and re-heating up to the final temperature $T_{f}$. Experimental data are depicted in Figure 1 which shows that the DSC scans for the first and second heating coincide. Figure 1 reveals that the glass transition temperature $T_{g}$ equals 151 °C, the crystallization temperature $T_{c}$ is 293 °C, and the melting temperature $T_{m}$ equals 339 °C. These values are in good agreement with observations in DSC tests on PEEK 90G ($T_{g}=155$, $T_{c}=317$, $T_{m}=345$ °C) [14] and PEEK 450G ($T_{g}=158$, $T_{m}=341$ °C) [10], as well as with the data in DMA test on PEEK 15G ($T_{g}=144$ °C) [11].

Mechanical tests were performed by means of a universal testing machine Instron–5568 equipped with a thermal chamber and an electro–mechanical sensor (Instron Static 2630113) for control of longitudinal strain in the active zone of samples. Tensile force was measured by a 50 kN load cell. The engineering stress σ was calculated as the ratio of axial force to the cross-sectional area of specimens in the undeformed state.

The experimental program included three series of tests at temperatures *T* ranging from room temperature to 180 °C. Each test was conducted on a virgin specimen. For each deformation program, tests were repeated three times of different samples to assess repeatability of measurements. The accuracy of measurements is estimated in Supplementary Material (Figures S1–S4) where experimental data are depicted (with their standard deviations) in selected uniaxial tensile tests, relaxation tests, and creep tests together with results of numerical analysis.

**Figure 2:**
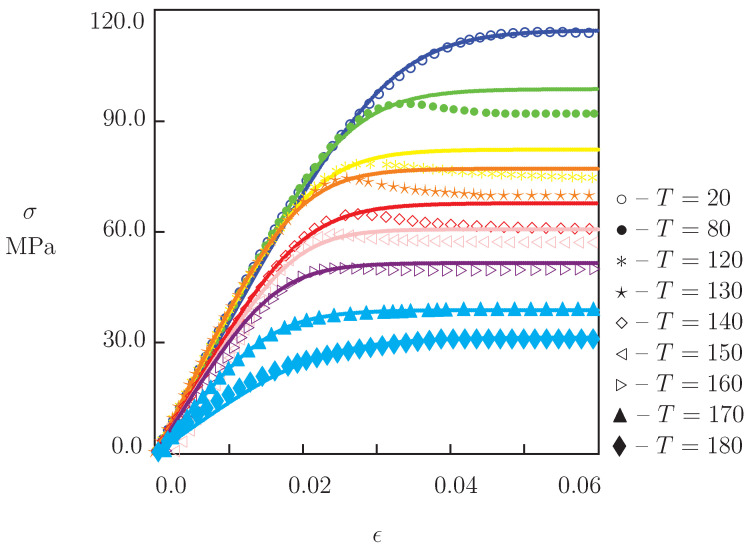
Stress σ versus strain ϵ. Symbols: experimental data in tensile tests with strain rate $\dot{\epsilon }=3.1\times 10^{-3}$ s${}^{-1}$ at temperatures *T* °C. Solid lines: results of simulation.

The first series involved uniaxial tensile tests with a cross-head speed of 20 mm/min (which corresponded to the strain rate $\dot{\epsilon }=3.1\times 10^{-3}$ s${}^{-1}$) up to breakage of specimens. The experimental stress–strain diagrams at temperatures T=20, 80, 120, 130, 140, 150, 160, 170 and 180 °C are depicted in Figure 2, where tensile stress σ is plotted versus engineering strain ϵ. We confine ourselves to the interval 0≤ϵ≤0.06 for necking of specimens occur under stretching in the post-yield region at temperatures below the glass transition point $T_{g}$, whereas we focus on the analysis of homogeneous uniaxial deformation with small strains.

**Figure 3:**
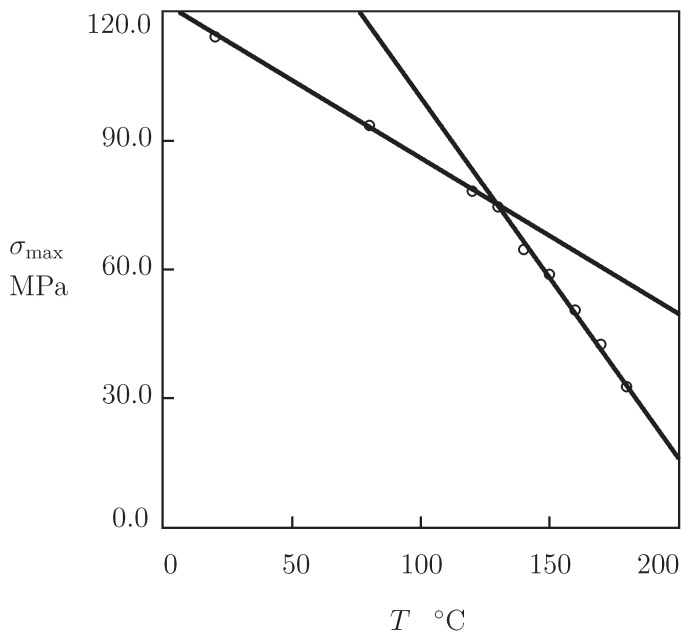
Tensile strength $\sigma_{\max}$ versus temperature *T*. Circles: experimental data in tensile tests with strain rate $\dot{\epsilon }=3.1\times 10^{-3}$ s${}^{-1}$. Solid lines: approximation of the data by Equation (1).

For each set of data, the maximum stress $\sigma_{\max}$ on the stress–strain curve was measured and associated with the yield stress. The effect of temperature on $\sigma_{\max}$ is illustrated in Figure 3, where $\sigma_{\max}$ is plotted versus *T*. With reference to [54], the data are approximated by the linear equation

(1) $$\sigma_{\max}=\sigma_{\max0}-\sigma_{\max1}T,$$

with the coefficients calculated by the least-squares technique. Figure 3 shows good agreement between the observations and their approximation by Equation (1) with different coefficients below and above the glass transition temperature $T_{g}$.

Our findings are in accord with observations on PEEK 450G reported in [10,21], which revealed changes in slope of the dependence $\sigma_{\max}\left(T\right)$ in the interval of temperatures between 135 and 140 °C.

**Figure 4:**
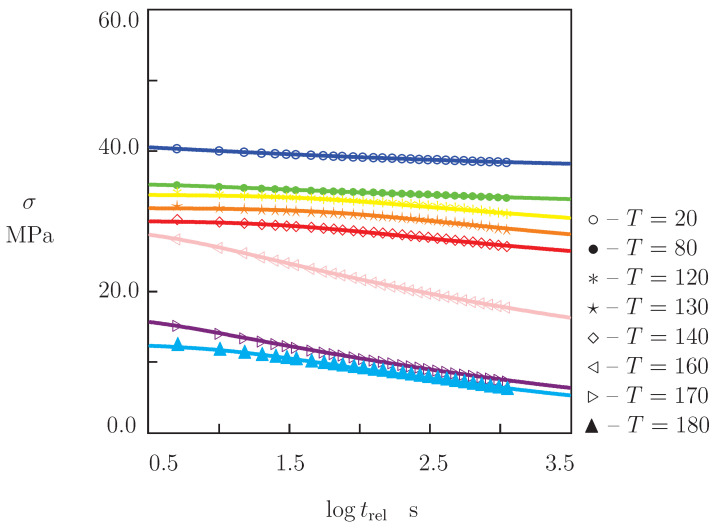
Stress σ versus relaxation time $t_{\mathrm{rel}}$. Symbols: experimental data in tensile relaxation tests with strain $\epsilon_{0}=0.01$ at temperatures *T* °C. Solid lines: results of simulation.

The other series of experiments involved tensile relaxation tests with a fixed strain $\epsilon_{0}=0.01$. In each test, a specimen was stretched with a cross-head speed of 20 mm/min up to the strain $\epsilon_{0}$. Afterwards, the strain was preserved constant, and the tensile stress σ was monitored as a function of time *t*. Following the protocol ASTM E-328, a duration of 20 min was chosen for the short-term relaxation tests. Experiments were carried out at temperatures T=20, 80, 120, 130, 140, 150, 160, 170 and 180 °C. Selected relaxation curves are reported in Figure 4, where tensile stress σ is plotted versus relaxation time $t_{\mathrm{rel}}=t-t_{0}$ ($t_{0}$ stands for the time needed to stretch samples up to the strain $\epsilon_{0}$). Following common practice, observations are presented by means of the semi-logarithmic plots with $\log=\log_{10}$.

In the last series of experiments, tensile creep tests were performed with various tensile stresses $\sigma_{0}$ at various temperatures *T*. In each test, a specimen was stretched with a cross-head speed of 20 mm/min up to the required stress $\sigma_{0}$. Afterwards, the stress was preserved constant, and an increase in strain ϵ was monitored a function of time *t*. Following the protocol ASTM D-2990, a duration of 20 min was chosen for the short-term creep tests.

**Figure 5:**
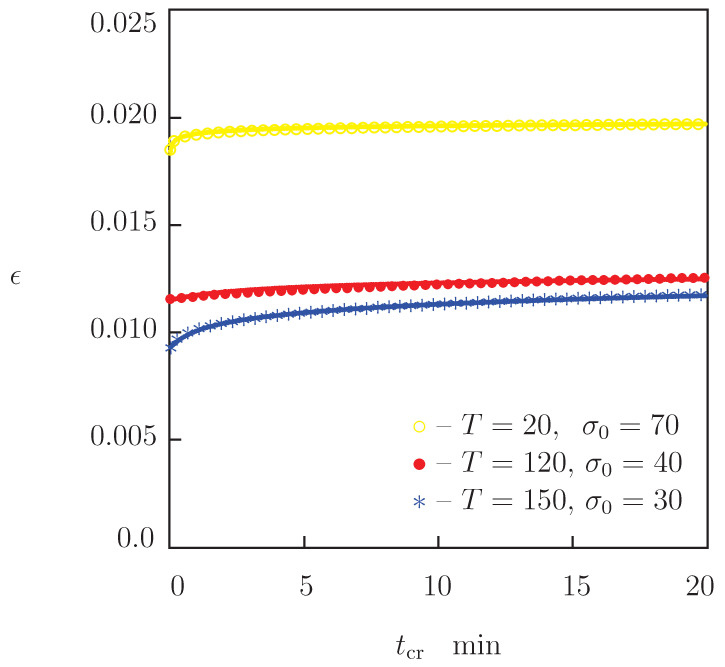
Strain ϵ versus creep time $t_{\mathrm{cr}}$. Symbols: experimental data in creep tests with various stresses $\sigma_{0}$ MPa at temperatures *T* °C. Solid lines: results of simulation.

**Figure 6:**
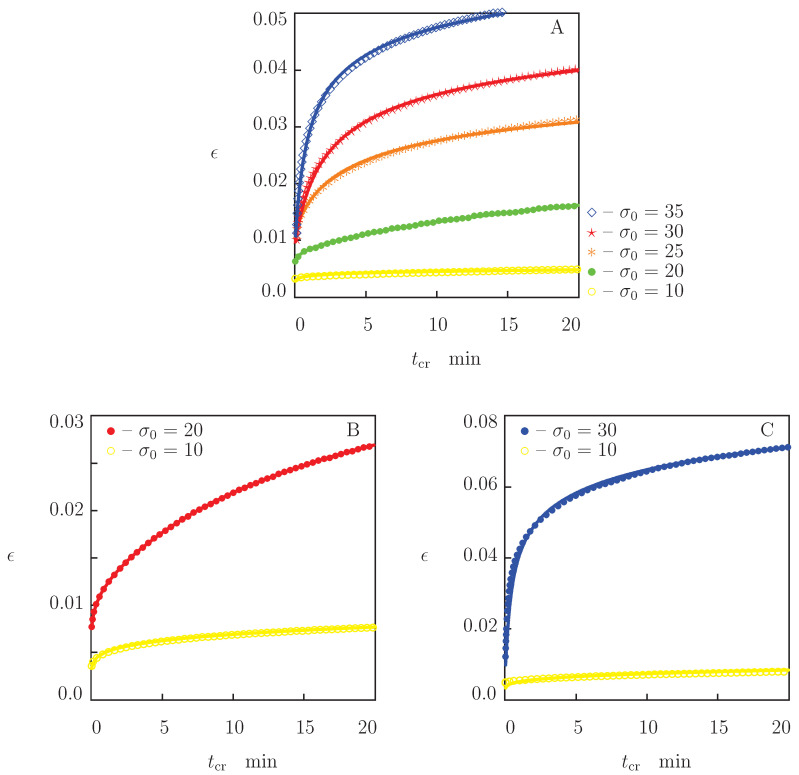
Strain ϵ versus creep time $t_{\mathrm{cr}}$. Circles: experimental data in creep tests with various stresses $\sigma_{0}$ MPa at temperatures T=160 °C (**A**), T=170 °C (**B**) and T=180 °C (**C**). Solid lines: results of simulation.

Two types of creep tests were conducted. Experimental data in these tests are reported in Figure 5 and Figure 6, where tensile strain ϵ is plotted versus creep time $t_{\mathrm{cr}}=t-t_{0}$ ($t_{0}$ stands for the time needed to reach the stress $\sigma_{0}$ under stretching).

In experiments of the first type, tensile stresses $\sigma_{0}$ were chosen to be relatively low. Observations in these tests are used to validate our model in linear viscoelasticity. Creep curves in selected tests (with $\sigma_{0}=70$ MPa at T=20 °C, $\sigma_{0}=40$ MPa at T=120 °C and $\sigma_{0}=30$ MPa at T=150 °C) are depicted in Figure 5, and those with $\sigma_{0}=10$ MPa at T=160, 170 and 180 °C are presented in Figure 6.

Tests of the other type were performed with relatively large tensile stresses $\sigma_{0}$ to evaluate the viscoplastic flow under creep conditions at temperatures above $T_{g}$. Experimental data in these tests are reported in Figure 6, where creep diagrams are depicted at temperatures T=160 °C (with $\sigma_{0}=20$, 25, 30 and 35 MPa), T=170 °C (with $\sigma_{0}=20$ MPa) and T=180 °C (with $\sigma_{0}=30$ MPa).

The following conclusions are drawn from Figure 2, Figure 4, Figure 5 and Figure 6: (i) under tension, stress σ decreases monotonically with temperature *T*, (ii) relaxation of stresses is negligible at temperatures below 130 °C and becomes noticeable at temperatures above $T_{g}$, (iii) creep flow below the glass transition temperature is weak (an increase in ϵ in short-term creep tests does not exceed 0.5%), while this flow becomes pronounced in tests with relatively large stresses above $T_{g}$ (tensile strain grows by several times).

## 3. Results and Discussion

We now develop simple constitutive equations for the thermo–mechanical response of PEEK and determine adjustable parameters in these relations by matching the experimental data in Figure 2, Figure 4, Figure 5 and Figure 6.

### 3.1. Thermo–Elastoplasticity

Under tension with a constant strain rate $\dot{\epsilon }$, ductile failure (necking) of specimens is observed in the post-yield region. As the failure process is unstable, we study the mechanical behavior of PEEK under tensile deformation up to the points of maximum on the stress–strain diagrams in Figure 2. At all temperatures *T*, tensile stress reaches its ultimate value at strains ϵ below 0.05, which corresponds to the duration of stretching of about 16 s. Figure 4 shows that relaxation of stresses during this period does not exceed 7%. This implies that the viscoelastic effects can be disregarded in the analysis of the stress–strain diagrams, and the response of PEEK can be described within the theory of elastoplasticity.

According to this concept, the total strain ϵ under uniaxial deformation equals the sum of the elastic, $\epsilon_{e}$, and plastic, $\epsilon_{p}$, components:

(2) $$\epsilon =\epsilon_{e}+\epsilon_{p}.$$

The stress σ is connected with the elastic strain $\epsilon_{e}$ by the linear equation

(3) $$\sigma =E\epsilon_{e},$$

where the Young’s modulus *E* is treated as a function of temperature *T*. The plastic strain $\epsilon_{p}$ is connected with the stress σ by the flow rule

(4) $${\dot{\epsilon }}_{p}=A\sinh\left(B\sigma \right)\sigma^{2},$$

where *A* is a function of temperature *T*, *B* is a temperature-independent material parameter, and the superscript dot stands for the derivative with respect to time *t*. The multiplier $\sigma^{2}$ in Equation (4) provides the simplest version of the Bailey–Norton law (its presence means that the rate of plastic deformation is governed by the stored mechanical energy [41]). The multiplier sinh(Bσ) is introduced to avoid the use of a yield surface (plastic deformation is presumed to occur at any stress, but its rate is negligible at small stresses due to the properties of the hyperbolic sinus). Another explanation for this term is based on the Eyring theory of thermally activated processes, according to which *B* is proportional to the activation volume for cooperative motion of polymer chains [55].

Equations (2)–(4) provide constitutive relations in thermo–elastoplasticity of PEEK under uniaxial deformation. These equations involve two functions of temperature, E(T) and A(T), and a constant *B* to be found by approximation of experimental data in Figure 2.

We begin with matching observations in tensile tests at room temperature, calculate *E* by fitting the data at 0≤ϵ≤0.01, and determine *A* and *B* from the best-fit condition for the entire stress–strain diagram. Then, the coefficient B=0.03 MPa${}^{-1}$ is fixed, and the stress–strain curves at elevated temperatures are matched by means of two parameters, *E* and *A*, only. Each set of observations is approximated separately. Figure 2 shows an acceptable agreement between the data and results of simulation.

To evaluate the activation volume $V_{a}$ associated with the coefficient *B* and to compare its values with results of other studies, the theory of thermally activated processes is applied. According to this concept,

(5) $$B=\frac{V_{a}\sqrt{3}}{k_{B}T_{0}},$$

where $k_{B}$ is the Boltzmann constant, $T_{0}=293$ K stands for room temperature, and the coefficient $\sqrt{3}$ appears due to transformation of tensile stress into the equivalent shear stress. For *B* found by fitting observations in Figure 2, it follows from Equation (5) that $V_{a}=7.0\times 10^{-2}$ nm${}^{3}$. This value is substantially (by two to three orders of magnitude) lower than $V_{a}=1$ nm${}^{3}$ [16], $V_{a}=1$ to 7 nm${}^{3}$ [21], $V_{a}=3.4$ nm${}^{3}$ [35], and $V_{a}=12.6$ nm${}^{3}$ [30]. This difference can be attributed to the fact that the activation volumes were calculated in the above works as measures of sensitivity of the yield stress to changes in the strain rate [55]. This explains also large (by an order of magnitude) deviations between the reported values of $V_{a}$.

The situation changes drastically if we associate $V_{a}$ with volumes of holes measured by means of the positron annihilation lifetime spectroscopy (PALS) and diffusivity of gases (these two methods lead to similar results [56]). PALS measurements of free volume imply that $V_{a}=7.15\times 10^{-2}$ nm${}^{3}$ for PEEK specimens [57], and this parameter varies between $7.2\times 10^{-2}$ to $8.4\times 10^{-2}$ nm${}^{3}$ depending on degree of crystallinity [58]. Both estimates are in good accord with the value of $V_{a}$ obtained in our analysis of observations.

**Figure 7:**
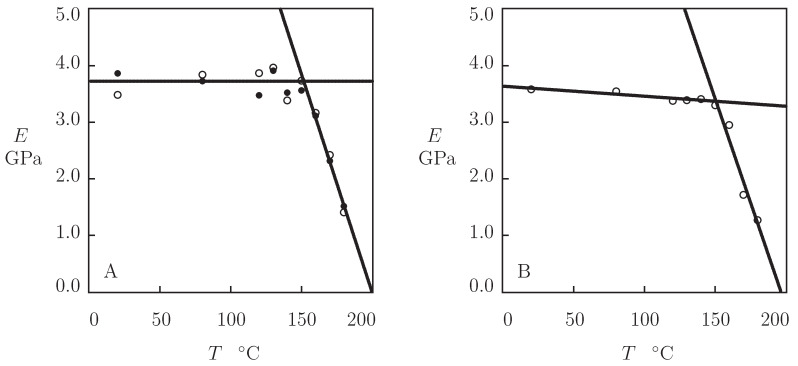
The Young’s modulus *E* versus temperature *T*. Symbols: (**A**)—treatment of observations in tensile tests (∘) and creep tests (•). (**B**)—treatment of observations in relaxation tests. Solid lines: approximation of the data by Equation (6).

**Figure 8:**
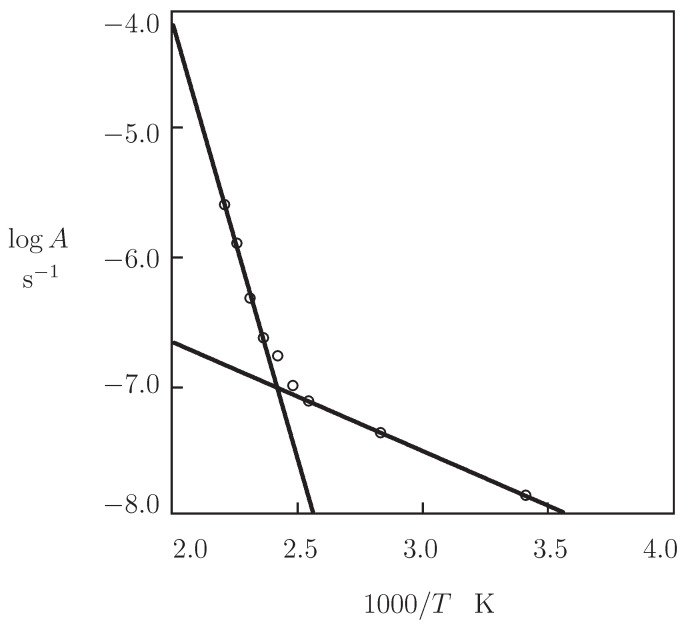
Coefficient *A* versus temperature *T*. Circles: treatment of observations in tensile tests. Solid lines: approximation of the data by Equation (7) with $E_{a}=16.7$ kJ/mol (low temperatures) and $E_{a}=133.3$ kJ/mol (high temperatures).

The effects of temperature *T* on the elastic modulus *E* and the rate of plastic flow *A* are illustrated in Figure 7 and Figure 8. By analogy with Equation (1), the data in Figure 7 are approximated by the linear function

(6) $$E=E_{0}-E_{1}T$$

with the coefficients accepting different values below and above the glass transition temperature $T_{g}$. Figure 7A shows that the modulus *E* remains practically constant below the glass transition temperature and decreases strongly above $T_{g}$. This behavior resembles that observed for the shear storage modulus [10] and tensile storage modulus [9,11] in DMA tests on PEEK.

The coefficient *A* is plotted versus reciprocal absolute temperature in Figure 8. The data are approximated by the Arrhenius dependence

(7) $$A=A_{0}\exp\left(-\frac{E_{a}}{RT}\right),$$

where $A_{0}$ is a pre-factor, *R* is the universal gas constant, and $E_{a}$ stands for the activation energy. Figure 8 shows that the observations are correctly described by Equation (7) when different activation energies are used below and above the glass transition temperature $T_{g}$.

### 3.2. Thermo–Viscoelasticity

The experimental data in tensile relaxation tests with a small strain $\epsilon_{0}=0.01$ at various temperatures *T* are described by the constitutive equations in linear viscoelasticity of semicrystalline polymers [59]. A polymer is thought of as a network with two types of chains: permanent (whose ends are bridged by covalent cross-links) and temporary (able to separate from their junctions and to merge with the network at random times being driven by thermal fluctuations). The heterogeneous network is composed of meso-domains with various activation energies for rearrangement. The rate Γ for separation of temporary chains from their junctions in meso-domains with a dimensionless activation energy *u* (normalized by $k_{B}T_{0}$) is governed by the Eyring equation

(8) Γ=γexp(−u),

where γ is a pre-factor. A quasi-Gaussian expression is adopted for the distribution function f(u) of meso-domains with various activation energies *u*,

(9) $$f\left(u\right)=f_{0}\exp\left(-\frac{u^{2}}{2\Sigma }\right)\;\left(u\geq 0\right).$$

The dimensionless parameter Σ characterizes inhomogeneity of an ensemble of meso-domains. The coefficient $f_{0}$ is determined by the normalization condition

(10) $$\int_{0}^{\infty }f\left(u\right)du=1.$$

Under uniaxial tension with an arbitrary deformation program ϵ(t), tensile stress σ(t) obeys the constitutive equation

(11) $$\sigma \left(t\right)=E\left[\epsilon \left(t\right)-\kappa \int_{0}^{\infty }\Gamma \left(u\right)f\left(u\right)du\int_{0}^{t}\exp\left(-\Gamma (u)(t-\tau )\right)\epsilon \left(\tau \right)d\tau \right],$$

where *E* stands for the Young’s modulus, and κ denotes the ratio of the number of temporary chains to the total number of chains per unit volume in the initial state. Unlike conventional (the Maxwell–Wiechert type) models for the linear viscoelastic response of polymers (involving a large number of material constants), Equation (11) is entirely determined by four parameters, *E*, κ, γ and Σ. With reference to [60], we suppose that Σ is independent of temperature, κ increases linearly with temperature and reaches its ultimate value κ=1 at relatively high temperatures $T>T_{g}$, and γ obeys the Arrhenius law. Equation (11) implies that, in tensile relaxation tests with a fixed strain $\epsilon_{0}$, the stress σ decreases with relaxation time $t_{\mathrm{rel}}$ following the pattern

(12) $$\sigma \left(t_{\mathrm{rel}}\right)=\sigma_{0}\left\{1-\kappa \int_{0}^{\infty }f\left(u\right)\left[1-\exp\left(-\gamma \exp\left(-u\right)t_{\mathrm{rel}}\right)\right]du\right\},$$

where

(13) $$\sigma_{0}=E\epsilon_{0}$$

stands for the stress at the beginning of the relaxation process.

Each set of data in Figure 4 is matched separately by means with Equation (12) with three parameters $\sigma_{0}$, κ and γ (since Σ is independent of temperature, we find its value Σ=7.0 by fitting observations at T=160 °C and use it at all temperatures *T* under consideration). The best-fit coefficient γ is determined by means of the nonlinear regression algorithm, while $\sigma_{0}$ and κ are determined by the least-squares technique. Given $\sigma_{0}$, the modulus *E* is calculated from Equation (13) and plotted versus temperature *T* in Figure 7B. This figure demonstrates that the data are described adequately by Equation (6) with different coefficients $E_{0}$ and $E_{1}$ below and above the glass transition temperature $T_{g}$.

The coefficients $E_{0}$ and $E_{1}$ found by approximation of experimental data in Figure 7A,B above $T_{g}$ coincide practically: the difference is less than 7% for $E_{0}$ and 5% for $E_{1}$. The discrepancies between the coefficients $E_{0}$ and $E_{1}$ calculated by matching observations in tensile and relaxation tests below $T_{g}$ are higher (but do not exceed 14%). They may be explained by local variations in thicknesses of specimens machined from an extruded sheet.

**Figure 9:**
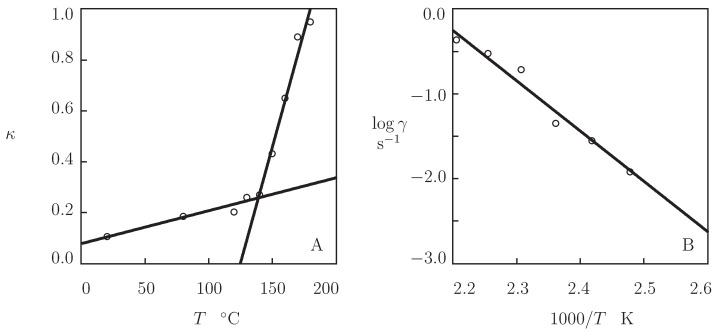
Parameters κ and γ versus temperature *T*. Circles: treatment of observations in relaxation tests. Solid lines: (**A**)—approximation of the data by Equation (14). (**B**)—approximation of the data by Equation (15) with $E_{a}=113.4$ kJ/mol.

The parameter κ is plotted versus temperature in Figure 9A. This figure demonstrates that κ increases monotonically with *T* (the growth of intensity of thermal fluctuations results in transformation of some permanent chains into transient chains). The influence of temperature *T* on κ is described by the linear equation

(14) $$\kappa =\kappa_{0}+\kappa_{1}T,$$

where $\kappa_{0}$ and $\kappa_{1}$ are calculated by the least-squares method. Figure 9A shows that Equation (14) describes correctly the function κ(T) when different coefficients $\kappa_{0}$, $\kappa_{1}$ are used below and above the glass transition temperature $T_{g}$.

The effect of temperature *T* on the rate of relaxation γ is illustrated in Figure 9B. The data (in the region of temperatures T≥120 °C) are approximated by the Arrhenius equation

(15) $$\gamma =\gamma_{0}\exp\left(-\frac{E_{a}}{RT}\right),$$

where $\gamma_{0}$ stands for a pre-factor, and $E_{a}$ denotes the activation energy. Comparison of Figure 8 and Figure 9B shows that the activation energies found by matching observations in tensile tests and relaxation tests in the high temperature region adopt similar values (deviations between $E_{a}=133.3$ kJ/mol in Figure 8 and $E_{a}=113.4$ kJ/mol in Figure 9B do not exceed 15 %).

**Figure 10:**
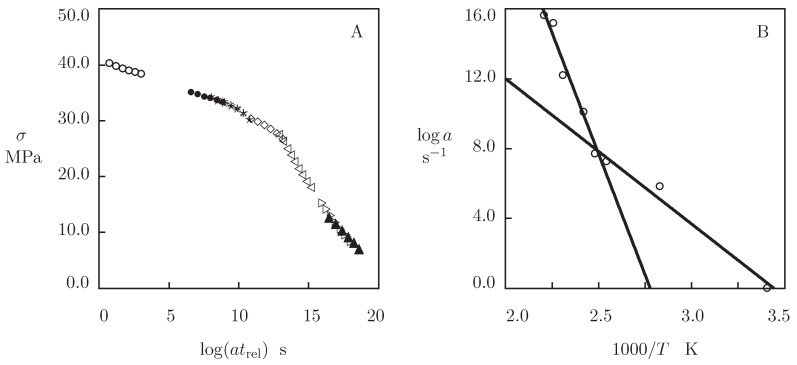
(**A**)—Stress σ versus relaxation time $t_{\mathrm{rel}}$. Symbols: master-curve at room temperature constructed by shift of experimental data in relaxation tests. (**B**)—Shift factor *a* versus temperature *T*. Circles: treatment of observations in relaxation tests. Solid lines: approximation of the data by Equation (16) with $E_{a}=159.4$ kJ/mol (low temperatures) and $E_{a}=531.3$ kJ/mol (high temperatures).

It is worth noting that these activation energies differ pronouncedly from the activation energies for α-relaxation reported in previous studies on the time-dependent response of PEEK ($E_{a}=377$ [38], $E_{a}=494$ [37] $E_{a}=582$ [61], $E_{a}=810$ to 1000 [9], $E_{a}=1094$ [39], and $E_{a}=1100$ kJ/mol [62]). To explain this difference, experimental data in relaxation tests are treated by the conventional method: all relaxation curves at elevated temperatures *T* are shifted horizontally (along the time-axis) to construct a master-curve at room temperature $T_{0}$. Figure 10A confirms that a smooth master-curve is formed by means of this technique. The shift factor *a* is plotted versus temperature *T* in Figure 10B. The data are approximated by the Arrhenius equation

(16) $$\log a=\log a_{0}+\frac{E_{a}}{RT},$$

where $a_{0}$ and $E_{a}$ are calculated by the least-squares technique.

Figure 10B shows good agreement between the data and predictions of Equation (16) with different coefficients below and above the glass transition temperature $T_{g}$. The activation energy in the low-temperature region $E_{a}=159.4$ kJ/mol is in accord with the activation energy $E_{a}=177$ kJ/mol for the β-relaxation in PEEK [9], whereas the activation energy in the high-temperature region $E_{a}=531.3$ kJ/mol is close to the activation energies $E_{a}=494$ kJ/mol [37] and $E_{a}=582$ kJ/mol [61] for the α-relaxation. These results demonstrate that the method based on the time-temperature superposition principle (shifts of observations in relaxation and dynamic mechanical tests) overly estimates the activation energies since this approach disregards evolution of *E* and κ with temperature *T*.

To confirm validity of our model for the linear viscoelastic behavior of PEEK, we apply Equation (11) to describe the time-dependent response of specimens in tensile creep tests and compare results of simulation with experimental data depicted in Figure 5 and Figure 6. Resolving Equation (11) with respect to ϵ, we find that the growth of tensile strain with time in a creep test with a constant stress $\sigma_{0}$ is governed by the equation

(17) $$\epsilon \left(t_{\mathrm{cr}}\right)=\epsilon_{0}+\kappa \int_{0}^{\infty }f\left(u\right)s\left(t_{\mathrm{cr}},u\right)du.$$

Here $t_{\mathrm{cr}}=t-t_{0}$, where $t_{0}$ is the moment when tensile stress reaches the required value $\sigma_{0}$,

(18) $$\epsilon_{0}=\frac{\sigma_{0}}{E}$$

is the strain at the beginning of the creep process, and the function $s(t_{\mathrm{cr}},u)$ obeys the differential equation

(19) $$\frac{\partial s}{\partial t_{\mathrm{cr}}}\left(t_{\mathrm{cr}},u\right)=\gamma \exp\left(-u\right)\left[\epsilon \left(t_{\mathrm{cr}}\right)-s\left(t_{\mathrm{cr}},u\right)\right],\;s\left(0,u\right)=0.$$

For each temperature *T* and stress $\sigma_{0}$, Equations (17)–(19) are integrated numerically by the Runge–Kutta method with the material constants γ, κ and Σ determined by matching observations in relaxation tests (Figure 9). To ensure good agreement between the observations in Figure 5 and Figure 6 and results of simulation, the coefficient *E* is treated as an adjustable parameter. Its best-fit values at various temperatures *T* are reported in Figure 7A. This figure shows that the Young’s moduli found by matching experimental data in tensile tests and creep tests coincide practically. Comparison of experimental data with results of simulation (Figure 5 and Figure 6) confirms the ability of the model (17)–(19) to predict the response of PEEK in short-term creep tests with small strains when material parameters are determined in relaxation tests.

### 3.3. Thermo–Viscoelastoplasticity

The time-dependent response of PEEK in creep tests with relatively high stresses $\sigma_{0}$ (beyond the interval of linear viscoelasticity) is described within the concept of viscoelastoplasticity [63]. In accord with Equation (2), the total strain ϵ is split into the sum of the viscoelastic strain, $\epsilon_{\mathrm{ve}}$, and plastic strain, $\epsilon_{p}$,

(20) $$\epsilon =\epsilon_{\mathrm{ve}}+\epsilon_{p}.$$

The viscoelastic strain $\epsilon_{\mathrm{ve}}$ is connected with tensile stress σ by Equation (11),

(21) $$\sigma \left(t\right)=E\left[\epsilon_{\mathrm{ve}}\left(t\right)-\kappa \int_{0}^{\infty }\Gamma \left(u\right)f\left(u\right)du\int_{0}^{t}\exp\left(-\Gamma (u)(t-\tau )\right)\epsilon_{\mathrm{ve}}\left(\tau \right)d\tau \right].$$

The evolution of the plastic strain $\epsilon_{p}$ with time *t* is governed by an analog of Equation (4),

(22) $${\dot{\epsilon }}_{p}=\bar{A}\sinh\left(\epsilon \right){\left(\sigma -\sigma_{b}\right)}^{m},\;\epsilon_{p}\left(0\right)=0,$$

where $\bar{A}$ stands for a pre-factor, the term sinh(ϵ) is introduced to avoid the growth of plastic strain at small strains (far below the yield point), and *m* is an exponent in the Bailey–Norton law. The back-stress $\sigma_{b}$ accounting for strain hardening under plastic flow [64] reads

(23) $$\sigma_{b}=E_{b}\epsilon_{p},$$

where $E_{b}$ is an analog of the elastic modulus.

Equations (20)–(23) together with Equations (8) and (9) provide a constitutive model in viscoelastoplasticity of semicrystalline polymers under uniaxial deformation. These relations involve four parameters, *E*, κ, γ and Σ, that characterize the linear viscoelastic response, and three extra coefficients, $\bar{A}$, $E_{b}$ and *m*. To reduce the number of parameters, we set m=7 (a typical value of the Bailey–Norton exponent) in matching experimental creep curves in Figure 6. The other two quantities, $\bar{A}$ and $E_{b}$, are considered as functions of temperature *T* only.

Under tensile creep with a fixed stress $\sigma_{0}$, Equation (21) takes a form similar to Equation (17),

(24) $$\epsilon_{\mathrm{ve}}\left(t_{\mathrm{cr}}\right)=\epsilon_{0}+\kappa \int_{0}^{\infty }f\left(u\right)s\left(t_{\mathrm{cr}},u\right)du,$$

while Equation (22) yields

(25) $$\frac{d\epsilon_{p}}{dt_{\mathrm{cr}}}=\bar{A}\sinh\left(\epsilon \right){\left(\sigma_{0}-\sigma_{b}\right)}^{m},\;\epsilon_{p}\left(0\right)=0.$$

The following procedure is applied to fit observations in Figure 6 at each temperature under consideration. First, the creep diagram with the highest stress $\sigma_{0}$ is approximated with the help of two parameters, $\bar{A}$ and $E_{b}$ (the quantities *E*, κ, γ and Σ are found by matching experimental data in the corresponding relaxation test). Afterwards, the quantities $\bar{A}$, $E_{b}$, *m*, κ, γ, Σ are fixed, and each remaining creep curve is fitted with the only coefficient *E*. We treat *E* as an adjustable parameter to account for deviations in thicknesses of specimens machined from an extruded sheet. Discrepancies between the best-fit values of *E* determined by matching creep curves with various $\sigma_{0}$ have the same order of magnitude as those between the data in tensile and creep tests in Figure 7 (they do not exceed 12 % at all temperatures).

**Figure 11:**
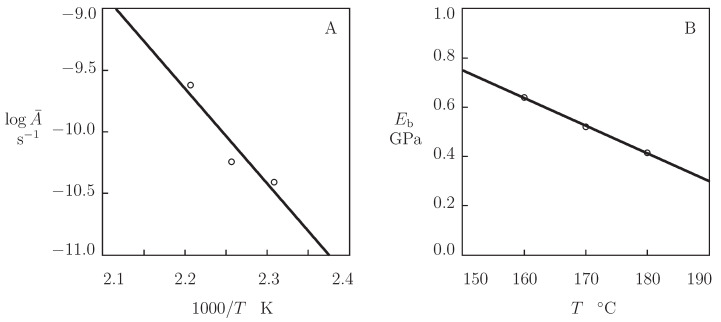
Parameters $\bar{A}$ and $E_{b}$ versus temperature *T*. Circles: treatment of observations in creep tests. Solid lines: (**A**)—approximation of the data by Equation (26) with $E_{a}=147.6$ kJ/mol. (**B**)—approximation of the data by Equation (27).

The effects of temperature on coefficients $\bar{A}$ and $E_{b}$ are illustrated in Figure 11.

The growth of the kinetic parameter $\bar{A}$ with *T* is described by the Arrhenius dependence,

(26) $$\bar{A}={\bar{A}}_{0}\exp\left(-\frac{E_{a}}{RT}\right).$$

Figure 11A shows an acceptable agreement between the data and their predictions by Equation (26) with the activation energy $E_{a}=147.6$ kJ/mol. The latter value is in agreement with the activation energy $E_{a}=133.3$ kJ/mol determined by matching observations in Figure 8 (the difference is less than 10 %).

The decay in $E_{b}$ with temperature is described by the equation analogous to Equation (6),

(27) $$E_{b}=E_{b0}-E_{b1}T,$$

where $E_{b0}$ and $E_{b1}$ are found by the least-squares method. Figure 11B demonstrates good agreement between the data and results of simulation. A similarity should be stressed between the effect of temperature on the Young’s modulus *E* and the modulus $E_{b}$ that characterize the back-stress. Above the glass transition temperature $T_{g}$, the dimensionless ratios $E_{1}/E_{0}$ adopt the values $5.09\times 10^{-3}$ (Figure 7A) and $5.01\times 10^{-3}$ (Figure 7B), whereas the ratio $E_{b1}/E_{b0}$ equals $4.62\times 10^{-3}$ (Figure 11B), which confirms that changes in *E* and $E_{b}$ with temperature are governed by the same physical mechanism.

## 4. Conclusions

Observations are reported on poly(ether ether ketone) KETRON 1000 PEEK in DSC tests (with a constant rate of 20 K/min under heating and cooling), as well as in uniaxial tensile tests with constant strain rate $\dot{\epsilon }=3.1\times 10^{-3}$ s${}^{-1}$, relaxation tests with a constant strain $\epsilon_{0}=0.01$, and creep tests with various stresses $\sigma_{0}$ (ranging from 10 to 70 MPa) at temperatures ranging from room temperature to 180 °C.

Constitutive equations are developed for the thermo–elastoplastic, thermo–viscoelastic and thermo–viscoelastoplastic responses of PEEK under uniaxial deformation with small strains. An advantage of these relations is that they involve relatively small numbers of adjustable parameters (three for the elastoplastic behavior, four for the linear viscoelastic response, and an extra three for the viscoelastoplastic flow). Good agreement is demonstrated between the experimental data and results of simulation.

Analysis of the effect of temperature on the rate of elastoplastic deformation *A*, the relaxation rate γ, and the rate of viscoplastic flow $\bar{A}$ reveals that the growth of these quantities with *T* above the glass transition temperature $T_{g}$ obeys the Arrhenius law with similar activation energies $E_{a}$ (in the range between 113 and 148 kJ/mol).

The study of the elastoplastic and viscoelastoplastic responses of PEEK shows that (i) the activation volume for plastic deformation coincides with the free volume found in PALS and diffusion tests and (ii) the elastic moduli *E* (stress) and $E_{b}$ (back-stress) decrease similarly with temperature. 

## Data Availability

The data that support the findings of this study are available from the corresponding author upon reasonable request.

## Supplementary Materials

The following are available online at https://www.mdpi.com/article/10.3390/polym13111779/s1, Figure S1: Stress σ versus strain ϵ. Symbols: experimental data in tensile tests at temperatures *T* = 20 (◦) and 170 (•) °C. Bars stand for the standard deviations. Solid lines: results of simulation, Figure S2: Stress σ_0_ versus relaxation time *t*_rel_. Symbols: experimental data in tensile relaxation tests with strain ϵ_0_ = 0:01 at temperatures *T* = 20 (◦) and 170 (•) °C. Bars stand for the standard deviations. Solid lines: results of simulation, Figure S3: Strain σ versus creep time *t*_cr_. Circles: experimental data in creep test with stress σ_0_ = 70:0 MPa at temperature *T* = 20 °C. Bars stand for the standard deviations. Solid line: results of simulation, Figure S4: Strain ϵ versus creep time tcr. Symbols: experimental data in creep tests with stresses σ_0_ = 10 (◦) and 20 (•) MPa at temperature *T* = 170 °C. Bars stand for the standard deviations. Solid lines: results of simulation.
