# Supplementary material for: Thermo-Mechanical Behavior of Poly(ether ether ketone): Experiments and Modeling
Source: Polymers (Basel). 2021 May 28;13(11):1779. doi: 10.3390/polym13111779 (PMC8199459; doi:10.3390/polym13111779)
Supplement: Supplementary file 1 [file polymers-13-01779-s001.zip › polymers-1219039-supplementary.pdf]

Supplementary material
Thermo-mechanical behavior of poly(ether ether ketone): experiments and modeling

A.D. Drozdov and J. deClaville Christiansen
Department of Materials and Production
Aalborg University
Fibigerstraede 16, Aalborg 9220, Denmark

S-1 Accuracy of fitting observations

To assess the ability of the model to describe observations on PEEK specimens, experimental data in selected tensile tests, relaxation tests and creep tests (at temperatures $T = 20$ and 170 °C) are presented with error bars (indicating standard deviations of measurements on different specimens) together with results of numerical analysis. Figs. S-1 to S-4 demonstrate good agreement between the data and results of simulation, which implies that the model predicts adequately observations of PEEK specimens.

S-2 Supplementary figures

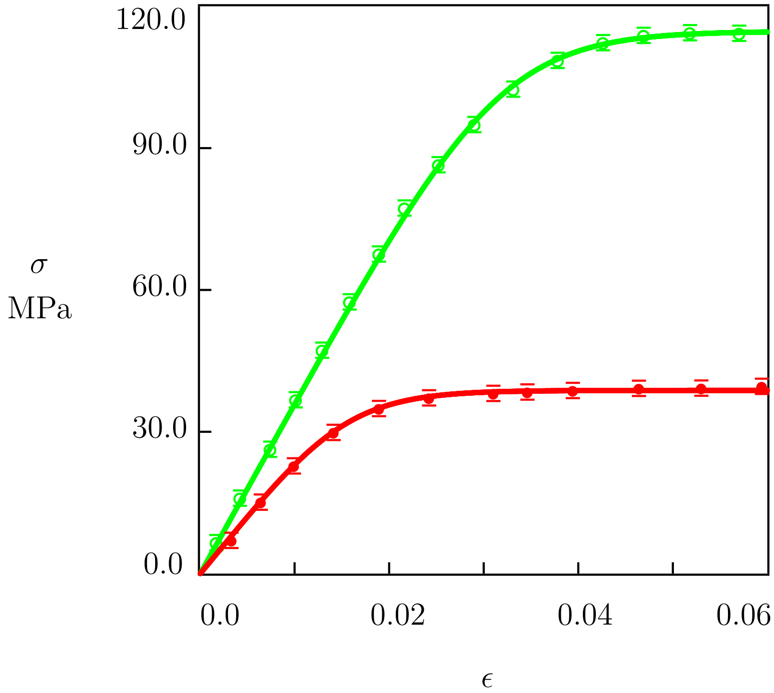

Figure S1: Stress σ versus strain ϵ. Symbols: experimental data in tensile tests at temperatures T = 20 ($\circ$) and 170 ($\bullet$) °C. Bars stand for the standard deviations. Solid lines: results of simulation.

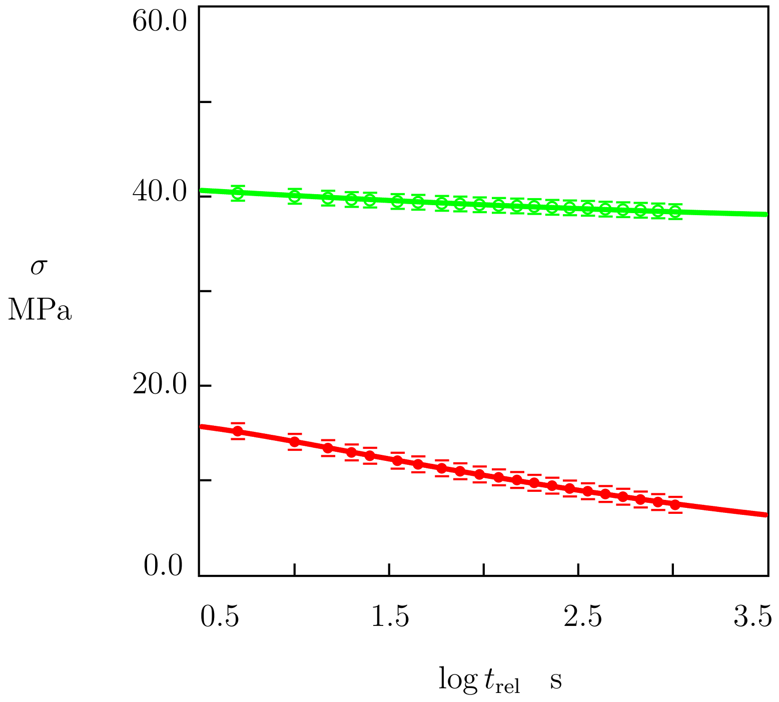

Figure S2: Stress σ versus relaxation time t_{rel}. Symbols: experimental data in tensile relaxation tests with strain $\epsilon_0 = 0.01$ at temperatures $T = 20$ ($\circ$) and 170 ($\bullet$) °C. Bars stand for the standard deviations. Solid lines: results of simulation.

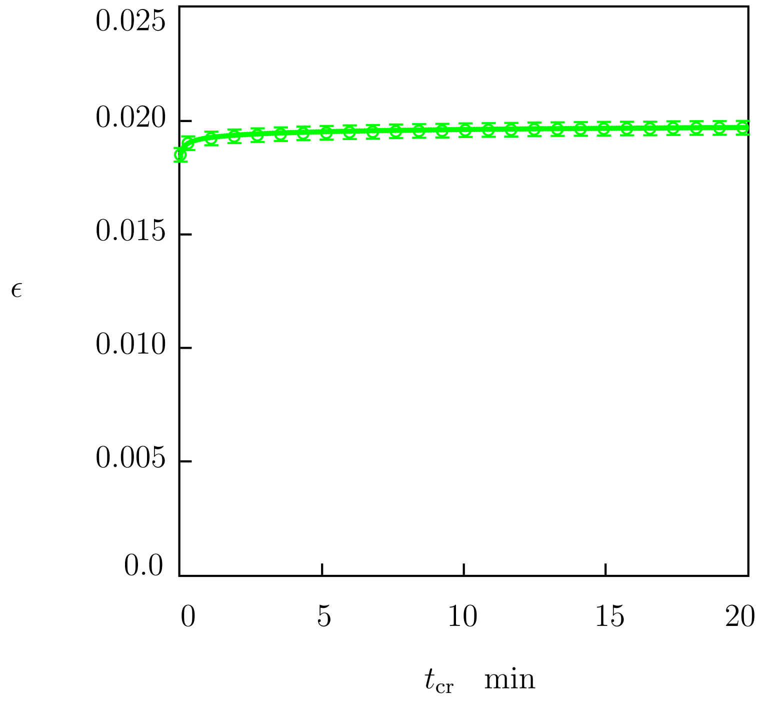

Figure S3: Strain ϵ versus creep time t_{cr}. Circles: experimental data in creep test with stress $\sigma_0 =$ 70.0 MPa at temperature $T = 20$ °C. Bars stand for the standard deviations. Solid line: results of simulation.

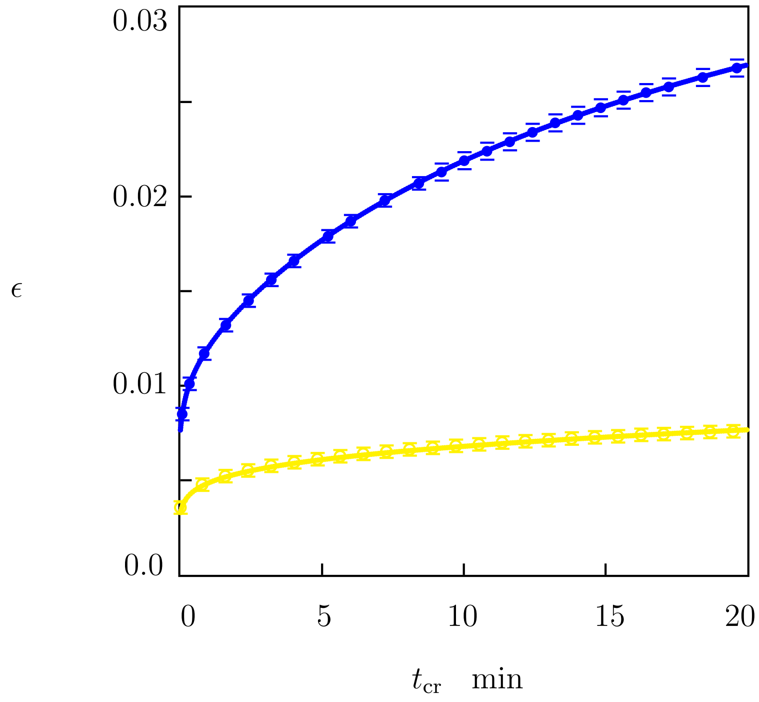

Figure S4: Strain ϵ versus creep time t_{cr}. Symbols: experimental data in creep tests with stresses $\sigma_0 = 10$ ($\circ$) and 20 ($\bullet$) MPa at temperature $T = 170$ °C. Bars stand for the standard deviations. Solid lines: results of simulation.
